# Differences in monocyte: lymphocyte ratio and Tuberculosis disease progression in genetically distinct populations of macaques

**DOI:** 10.1038/s41598-019-39819-6

**Published:** 2019-03-04

**Authors:** Laura Sibley, Karen Gooch, Alice Wareham, Susan Gray, Andrew Chancellor, Stuart Dowall, Simon Bate, Anthony Marriott, Mike Dennis, Andrew D. White, Philip D. Marsh, Helen Fletcher, Sally Sharpe

**Affiliations:** 1 National Infection Service, Public Health England, Porton Down, Wiltshire UK; 2 London School of Hygiene and Tropical Medicine, London, UK

## Abstract

Monocyte:lymphocyte ratio (M:L) has been identified as a risk factor in development of TB disease in children and those undergoing treatment for HIV in co-infected individuals. Retrospective analysis was performed using M:L data collected from TB modelling studies performed in Rhesus macaques of Indian genotype (RM), cynomolgus macaque of Chinese genotype (CCM) and cynomolgus macaque of Mauritian genotype (MCM), which found that the more susceptible populations (RM and MCM) had higher M:L ratios than the least susceptible population (CCM). Following *Mycobacterium tuberculosis* exposure, significant increases in M:L ratio were observed in susceptible RM and MCM within 12 weeks of TB infection, whereas M:L in CCM remained stable, suggesting that changes in M:L ratio may also act as a biomarker of TB disease progression. The frequency of PPD-specific interferon gamma (IFNγ) secreting cells (SFU) were compared, with the more susceptible macaque populations showing an association between M:L and IFNγ SFU frequency. Investigation of the genes associated with monocyte-derived antigen presenting cells revealed differences between RM and CCM, highlighting differences in their monocyte populations, as well as overall M:L ratio. Differences in M:L ratio between macaque populations could be used to explore immunological mechanisms in susceptible populations that would complement human population studies.

## Introduction

The ratio of monocytes to lymphocytes defined in peripheral blood has been associated with the outcome of several diseases; including several types of cancer^1,2^, coronary heart disease^3^, Hepatitis B^4^, HIV^5^ and Tuberculosis (TB)^5,6^, among others. In cases of cancer, it has been found that a chronic inflammatory situation, characterised by a high M:L is predictive of treatment failure and advancement of disease^2^. Furthermore, patients with different stages of cancer have different M:L ratio, with those with stage III-IV cancer having a higher M:L ratio than those with stage I-II^1^.

The importance of the M:L in TB disease progression was first suggested in the 1920’s in rabbits infected with TB^7,8^. In animals where disease was severe, Cunningham and Sabin observed that the number monocytes in the peripheral blood increased only in animals where disease progressed. In animals that controlled the disease, numbers of lymphocytes and monocytes in the periphery increased following infection maintaining the ratio with a greater proportion of lymphocytes.

More recently, a high M:L has been shown to distinguish persons with active and latent TB from uninfected persons^5^, and been used to predict risk of developing TB in infants^6^. M:L has been found to reduce after treatment for HIV, and corresponded with improvement in the patient’s condition^5^. Therefore, in humans it appears that the M:L shows promise as an indicator of risk of developing active TB and could facilitate the targeting of preventative treatments/therapy for those who are defined as being at greater risk.

The non-human primate provides the most relevant model of human TB infection because the disease and immune response developed are similar^9^. Rhesus macaques of Indian origin (RM), cynomolgus macaques of Chinese origin (CCM) and cynomolgus macaques of Mauritian origin (MCM) are commonly used to study tuberculosis infection.

Monocyte and lymphocyte number and frequencies have been analysed retrospectively from data generated at PHE NIS over the last ~15 years from studies conducted with RM, CCM and MCM, and used to compare M:L ratios before and after infection with *Mycobacterium tuberculosis* (MTB). M:L was measured using three different methods, using two types of samples; the Hemavet haematology analyser, IDEXX haematology analyser and flow cytometry using whole blood samples, and flow cytometry applied to PBMCs.

The association of M:L with other changes in the mycobacterial specific immune response were investigated by using IFNγ ELISPOT and transcriptomics to analyse changes in gene signatures associated with cytokine expression and macrophage and dendritic cell signatures.

## Results

### Susceptibility of macaque populations to MTB

**Figure 1:**
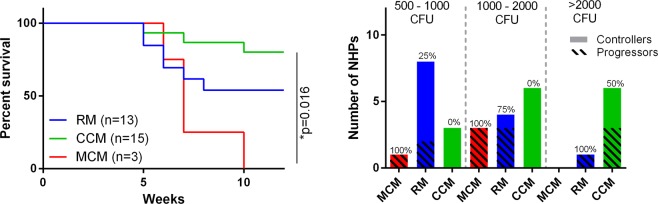
Comparison of genetically distinct populations of macaques in terms of control of TB disease. (**a**) Survival plot of RM, MCM and CCM following exposure to a presented dose in the range of 500–5000 CFU of Mycobacterium tuberculosis (MTB) Erdmann strain. RM (blue), CCM (green), MCM (red). (**b**) Distribution of NHPs from different populations across three different dose ranges; 500–1000 CFU, 1000–2000 CFU and >2000 CFU. RM > 2000 CFU n = 1, RM 1000–2000 CFU n = 4, RM 500–1000 CFU n = 8, CCM > 2000 CFU n = 6, CCM 1000–2000 CFU n = 6, CCM 500–1000 CFU n = 3 MCM 1000–2000 CFU n = 3, MCM 500–1000 CFU n-1. Black stripes = progressors, Clear bars = Controllers. Percentage of population at each dose range that had progressive TB disease displayed above each bar.

Data were combined from several studies, where RM and MCM (susceptible) and CCM (controllers) were infected following exposure to a presented dose of MTB (Erdmann) within the range 500–5000 CFU and shown in three dose levels; 500–1000 CFU, 1000–2000 CFU and >2000 CFU to examine associations with dose (Fig. 1a). MCM were only given the lower two dose ranges and all developed disease that met humane end point criteria before the end of the 12 week study (Fig. 1b). Overall, 50% of RM were unable to control TB infection, and this was across all doses, although outcome was improved with the lowest dose (500–1000 CFU) (Fig. 1b). Across all doses, 20% of CCM were unable to control TB infection and this was only at the highest doses of over 2000 CFU, between 500–2000 CFU, all CCM were able to control MTB infection (Fig. 1b).

### M:L before MTB infection

**Figure 2:**
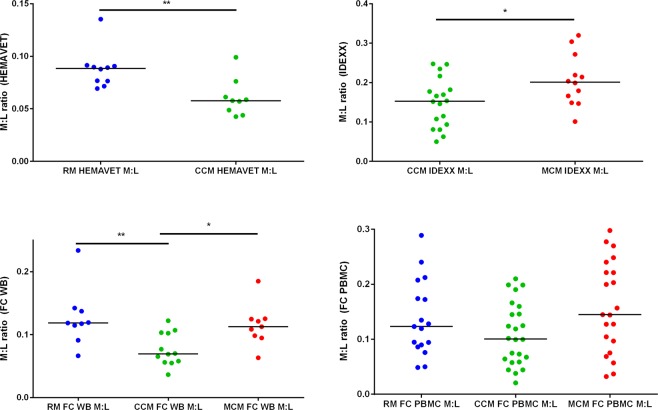
M:L derived from data using four techniques; (**a**) HEMAVET or (**b**) IDEXX haematology analysers and flow cytometry (FC) cell surface staining of (**c**) whole blood (WB) or (**d**) PBMCs to compare differences between the species. RM (blue), CCM (green) and MCM (red). Median shown. Mann-Whitney U test performed. HEMAVET RM n = 10, CCM n = 0, IDEXX CCM n = 18, MCM n = 12, FC WB RM n = 9, CCM n = 12, MCM n = 9, FC PBMC RM n = 17, CCM n = 24, MCM n = 23. *p = < 0.05 **p = < *0.005*.

M:L ratio was calculated for each of the three macaque populations from individuals enrolled in a variety of studies (Table 1) using data generated using either haematology, or flow cytometry (FC) analysers applied to whole blood (Hemavet and IDEXX haematology analysers and FC), or PBMC (FC only) (Fig. 2). M:L derived from three data sets showed M:L to be significantly higher in MCM (progressors) than CCM (controllers) (IDEXX *p =* 0.04*) (Fig. 2a–c). Following application of the Bonferroni correction for multiple comparisons to the data sets generated using FC (Fig. 2c,d) the p value required to meet significance reduced to 0.025. The difference in M:L between species revealed using M:L derived from FC WB (Fig. 2c) met the corrected requirement for significance, and a similar non-significant trend was seen in the FC PBMC data set (FC WB *p =* 0.01*, FC PBMC *p *=* 0.04*). M:L was significantly higher in RM (progressors) than in CCM in two of the data sets (HEMAVET **p =* 0.004*, FC WB **p =* 0.004* (FC WB using Bonferroni correction)). FC PBMC data showed the same trend but was not significant.

**Table 1:** Numbers of macaques of different populations used for each type of analysis.

	Data set size (Number of macaques)					
	Haematology		Flow cytometry		ELISPOT	DGE
	IDEXX	Haemavet	WB	PBMC		
**Prior to mycobacterial exposure**						
RM	0	10	9	17	0	0
CCM	18	9	12	24	0	0
MCM	12	0	9	23	0	0
**Post MTB exposure**						
RM	0	0	9	0	9	4
CCM	0	0	9	0	9	6
MCM	0	0	9	0	9	0

RM; rhesus macaque; CCM: Chinese cynomolgus macaque; MCM: Mauritian cynomolgus macaque; WB: whole blood; PBMC: peripheral blood mononuclear cells; DGE differential gene expression.

### M:L post-MTB infection

**Figure 3:**
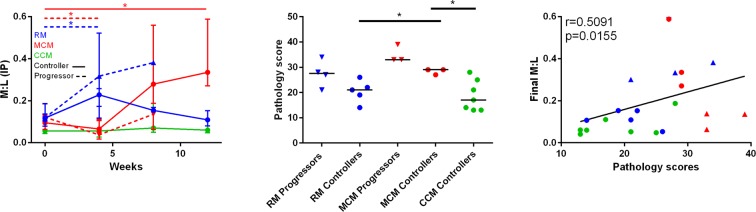
M:L ratios and total pathology scores determined in RM (blue), MCM (red) and CCM (green) following aerosol exposure to *M. tuberculosis* at a presented dose in the range 250–1400 CFU. (**a**) M:L ratio of progressors and controllers for infected RM, MCM and CCM group medians with interquartile range at determined at intervals after MTB aerosol exposure. RM progressors at week 4 were significantly different from week 0 (dotted blue horizontal line) (*p = *0.02)*, MCM progressors were significantly lower than week 0 (dotted horizontal red line) (*p = *0.02*) and MCM controllers were significantly lower than week 0 at week 12 (red horizontal line) (*p = *0.02*) as measured using Mann Whitney U-test (*p=<0.05). RM n = 9, MCM n = 6, CCM n = 7. (**b**) Total pathology scores of progressors (triangles) and controllers (circles) of infected RM, MCM and CCM. MCM controllers had significantly higher total pathology scores than both RM controllers (*p = *0.04*) and CCM controllers (*p = *0.03*) as measured using Mann Whitney U-test. (*p=<0.05). RM controllers n = 5, RM progressors n = 4, MCM controllers n = 3, MCM progressors n = 3, CCM controllers n = 7. (**c**) Spearman’s rank correlation of the final M:L value for each animal (i.e. week 12 or when they reached humane end point criteria) versus the pathology scores *r* = *0.51, p* = *0.016*. Controllers = circles, progressors = triangles, RM = blue, MCM = red, CCM = green. *p=<0.05. RM controllers n = 5, RM progressors n = 4, MCM controllers n = 3, MCM progressors n = 3, CCM controllers n = 7.

The M:L was evaluated at intervals after MTB challenge (Fig. 3) in groups of rhesus, Chinese and Mauritian cynomolgus macaques (Table 1) that either controlled disease (‘controllers’) or developed progressive disease (‘progressors’) during the first 12 weeks of infection. Infection with MTB did not significantly perturb the M:L measured in CCM (controllers), whereas an increase in the M:L compared to pre-infection levels, was revealed in both the RM and MCM post-infection (Fig. 3). A peak in M:L ratio occurred in RM four weeks after infection whereas the increase was slower in MCM and still rising at 12 weeks.

The M:L ratio was compared between controllers and progressors and found that there was no significant difference at individual time points for either RM or MCM. When comparing pre-challenge and post-challenge M:L, the progressor RM had significantly higher M:L at week four than pre-infection (*p =* 0.02*), but not at other time points. In contrast, the MCM that controlled disease, possessed an M:L four weeks after infection that was significantly lower (*p =* 0.02*) than the levels measured pre-infection. By week 12, the remaining ‘controller’ MCM group showed a median M:L that was increased significantly compared to pre-infection (*p =* 0.02*). The M:L measured in CCM, who controlled disease during the 12 week study period, was consistently lower than those measured in than the RM and MCM populations across the time course. When comparing the pathology scores of controllers and progressors in the three populations, the M:L for the MCM controllers group, were significantly higher than the RM and CCM controllers groups (RM *p =* 0.04*, CCM *p =* 0.03*) suggesting that the MCM controllers were not controlling disease as well as the others, and if the study had been longer disease may have progressed. When the M:L at the last time point for each animals was correlated with total pathology score, there was a significant correlation between high M:L and high pathology score (*r *= 0.5, *p =* 0.02* Spearman’s rank correlation).

### M:L and the immune response

**Figure 4:**
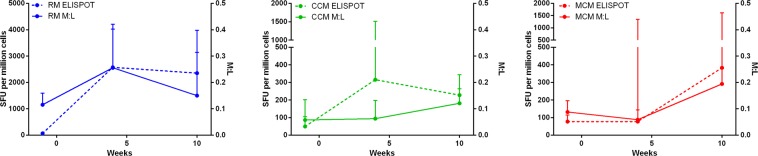
PPD-specific IFNγ SFU measured by ELISPOT overlaid with M:L ratio for (**a**) RM (blue) n = 9, (**b**) CCM (green) n = 7 and (**c**) MCM (red) n = 6 post-infection with MTB. Median shown with standard deviation.

The MTB-specific immune response induced following infection was measured in each of the three macaque populations using a PPD-specific IFNγ ELISPOT assay. In order to investigate putative relationships between M:L and the frequency of PPD-specific IFNγ SFU, the profiles were overlaid (Fig. 4). In both the RM and MCM susceptible populations, the frequency of PPD-specific IFNγ SFU increased in parallel with the M:L. The frequency of PPD-specific IFNγ SFU in controller CCM was not associated with the M:L ratio.

### Differential gene expression of genes associated with the cytokine response, and DCs, M1 and M2 macrophages e to MTB infection

**Figure 5:**
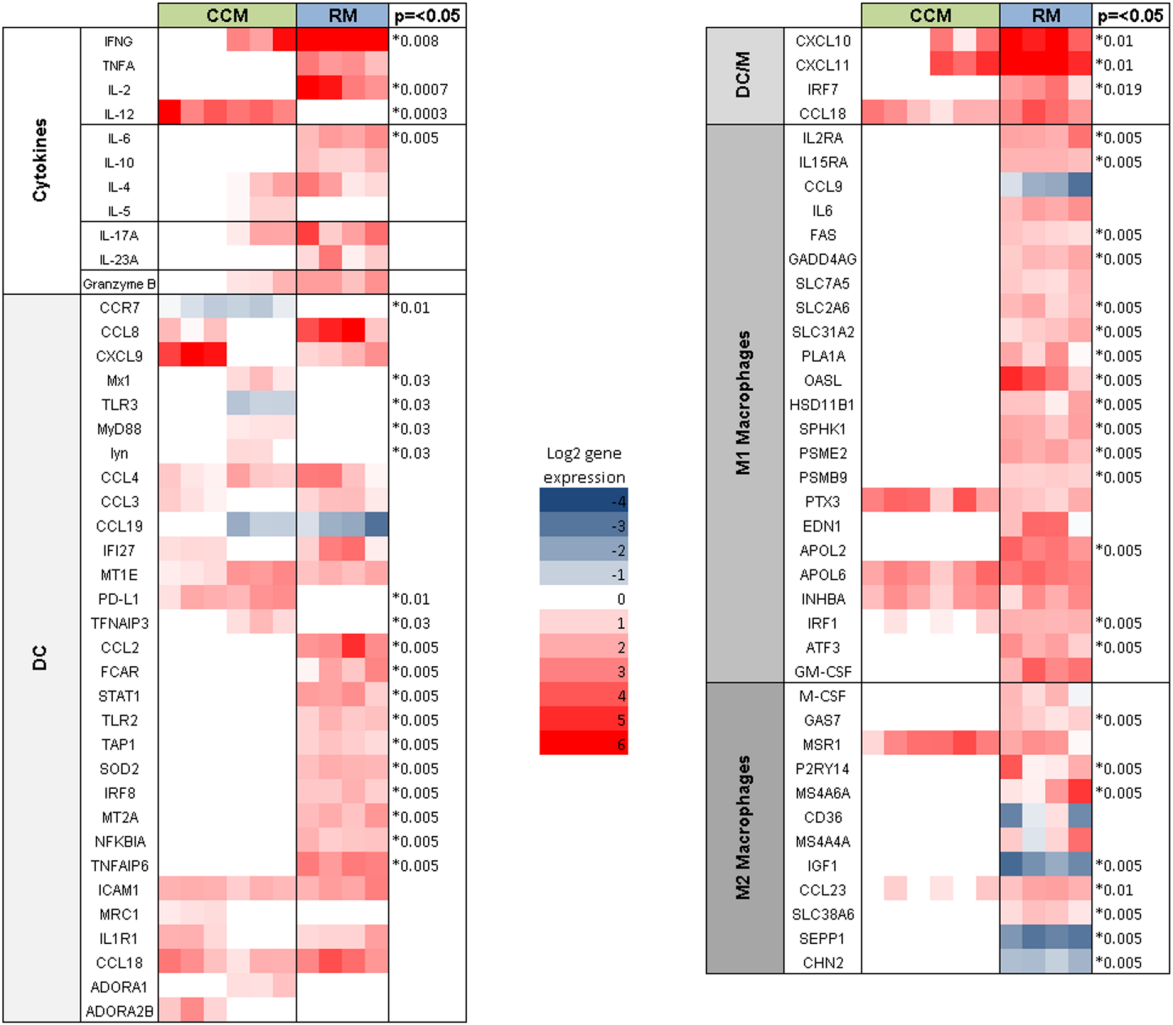
Log2 differential gene expression of genes associated with cytokines, DCs and M1 and M2 macrophages, by PBMCs collected from RM (blue, n = 4) and CCM (green, n = 6) six weeks post-infection with MTB and stimulated with PPD. Mann-Whitney U test * < p = 0.05. IFNγ *p = *0.008*, IL-2 *p = *0.0007*, IL-12 *p = *0.0003*, IL-6 *p* = *0.005*, GAS7 *p = *0.005*, IRF7 *p = *0.19*, ATF3 *p = *0.005*, IRF1 *p = *0.005*, CHN2 *p = *0.005*, SEPP1 *p = *0.005*, APOL2 *p = *0.005*, PSMB9 *p = *0.005*, PSME2 *p = *0.005*, SPHK1 *p = *0.005*, HSD11B1 *p = *0.005*, OASL *p = *0.005*, PLA1 *p = *0.005*, SLC38A6 *p = *0.005*, SLC31A2 *p = *0.005*, SLC2A6 *p = *0.005*, GADD4AG *p = *0.005*, FAS *p = *0.005*, IGF1 *p = *0.005*, CCL23 *p = *0.01*, CXCL10 *p = *0.01*, CXCL11 *p = *0.01*, MS4A6A *p = *0.005*, P2RY14 *p = *0.005*, IL15RA *p = *0.005*, IL2RA *p = *0.005*, CCR7 *p = *0.01*, Mx1 *p = *0.03*, TLR3 *p = *0.03*, MyD88 *p = *0.03*, lyn *p = *0.03*, PD-L1 *p = *0.01*, TNFAIP3 *p = *0.03*, CCL2 *p = *0.005*, FCAR *p = *0.005*, STAT1 *p = *0.005*, TLR2 *p = *0.005*, TAP1 *p = *0.005*, SOD2 *p = *0.005*, IRF8 *p = *0.005*, MT2A *p = *0.005*, NFKB1A *p = *0.005*, TFNAIP6 *p = *0.005*.

Genes encoding for relevant cytokines following PPD stimulation measured using differential gene expression of PBMCs differed between RM and CCM after infection with MTB **(**Fig. 5**)**. IFNγ expression was significantly higher in RM (*p =* 0.008*), as was IL-2 (*p =* 0.0007*). IL-12 gene expression was significantly higher in CCM (*p =* 0.0003*). TNFα, which is also a Th1 defined cytokine was higher in RM. Cytokines associated with Th17, Th2 and cytotoxic T-cells (Tc) showed a trend for higher expression in RM than CCM. To further characterise the antigen presenting cells in RM and CCM, genes associated with the development of monocyte derived DCs as defined by Schinnerling *et al*.^10^ and those associated with M1/M2 macrophages from Martinez *et al*.^11^ were examined in more detail. Differential Gene Expression of RM and CCM showed increased expression of genes associated with DC, but the RM population showed a higher expression of genes associated with M1 and M2 macrophages.

Differences in the gene expression of 24 genes associated with cytokine production or DC including IFNγ and MyD88 were noted with expression patterns forming two clusters within the CCM population investigated dependent on ability to control TB-induced disease (Fig. 5), between individuals in which disease progressed to meet the humane end point criteria before the end of the study (Fig. 5, columns 1–3), compared to individuals that were able to control disease (Fig. 5, columns 4–6). Investigation of the rhesus macaque population revealed the differential regulation of five genes including PLA1A and M-CSF between individuals that were able to control disease progression during the study (Fig. 5, columns 10) and those in which disease progressed to meet the humane end point criteria before the end of the planned study period (Fig. 5, columns 7–9).

## Discussion

For our analysis of the M:L ratio, we used data collected using either haematology or flow cytometry based approaches dependent on the materials and analysers available when each study was performed. Early NHP studies carried out at PHE used a HEMAVET haematology analyser, which was replaced by an IDEXX haematology analyser for later studies. Evaluation of M:L using flow cytometry was applied to either whole blood or PBMC samples. This provided the opportunity to compare M:L data sets between studies, populations and techniques. We have demonstrated that within each technique it is possible to identify individuals with high or low M:L and that the hierarchies identified between species were consistent across methodologies. However significant differences in the absolute M:L values derived was seen between methods which is important because cut off values for the M:L, above which a patient is at risk of developing TB or some other disease have been previously defined and so must be method specific^2,5^.

Data collected before application of experimental interventions revealed a higher M:L in RM and MCM than in CCM across all methods used and the difference reached statistical significance with all approaches except that derived using flow cytometric analysis of stained PBMC. The difference in density of gradients used for cell isolation from the two macaque species may be responsible for the decreased sensitivity offered by this later technique or PBMC isolation and the manipulation process may have altered the overall populations to affect the ratio. The higher M:L ratio in RM and MCM appears to be associated with their increased susceptibility to disease, as defined by survival post-infection. It is noteworthy that in the cohort of 18 CCM who received aerosol doses towards the upper limit of dose range only three of the cohort of eighteen CCM developed progressive disease that met humane end point criteria, whereas the doses the RM and MCM were much lower and the development of progressive disease more frequent. Data from only three MCM was available for this comparison, as MCM were not used in further studies involving high dose challenge after the initial pilot study revealed the increased susceptibility of MCM to TB.

The M:L was investigated in macaques after infection with TB to determine the impact of infection, particularly whether M:L increased in line with observations from human studies where it has been demonstrated that individuals with active disease have a higher M:L ratio than healthy donors^12^. Overall, comparison of M:L between the populations post-infection suggested that progressive disease is associated with an increase in M:L ratio, as RM and MCM possessed a higher M:L than CCM. Differences in M:L were also identified between controllers and progressors within the RM population that substantiated that controllers had a lower M:L than progressors. However, MCM controllers and progressors differed to RM. MCM progressors initially had a lower M:L post-infection, however, there is also evidence to suggest that very low M:L is also a risk factor for developing TB^5^, and this population may be processing the infection in a different way. At later time points, the ‘controller’ MCM have a significantly higher M:L compared to baseline, and when the pathology scores were analysed, the pathology scores were significantly higher than RM and CCM controllers, suggesting that disease was in fact progressing in the MCM controllers and if the study had continued then they may have met humane end point criteria, and then supports the RM data and human data that a high M:L ratio is a biomarker for active disease.

The relationships between M:L and the wider immune response were explored further to ascertain the potential effect that an increased M:L has on the rest of the immune system. The interaction of macrophages and dendritic cells with T-cells through the presentation of antigen is crucial for development of an effective adaptive immune response. Monocytes differentiate to macrophages and dendritic cells that present antigen to T-cells and therefore increased numbers of monocytes could activate more T-cells.

The relationship between M:L and the T-cells was investigated using the PPD-specific IFNγ ELISPOT assay. IFNγ has long been considered essential for control of TB infection, and IFNγ gene disrupted mice are more susceptible to TB^13^, but IFNγ may not provide a good correlate of protection^14^. In the studies compared here, the increase in IFNγ producing cells did not correspond with an increased ability to control TB, as RM had the highest frequency of PPD-specific IFNγ SFU but was less able to control disease than CCM.

The frequency profile of PPD-specific IFNγ SFU suggested an association between M:L levels in the MCM and RM populations as IFNγ production increased at the same time points as M:L, but this did not occur in the CCM. We hypothesise that the increase in M:L in MCM and RM was indicative of an increase in monocyte numbers, which caused an increase in APCs, and therefore increased T-cell activation and IFNγ production. However, the lack of increase in M:L in the CCM, suggests the overall number of APCs did not increase, and therefore this species may possess an alternative mechanism to induce T-cell activation.

To further characterise the immune response downstream of the monocytes and determine the characteristics of the APCs that could account for the differences seen in M:L and ELISPOT profiles, cytokine gene expression was investigated.

Genes associated with conventional T-cell subsets; Th1, Th2, Th17 and Tc were identified from RM and CCM microarray data post TB infection. Th1 cytokines can also be associated with M1 macrophages and there were higher levels of IFNγ and TNFα expression associated with RM, in comparison to CCM. IL-12 was higher in CCM, which could be attributed to DCs and Th1 immune responses because of the pattern of expression of other cytokine genes. The Th2 genes could also be attributed to M2 macrophages and these were all upregulated in the RM.

However, as these cytokines could also be attributed to T-cells in the mixed populations of PBMCs, genes associated with DCs, M1 and M2 macrophages were identified from the literature for further examination^10,11^. Genes associated with DCs were expressed in both CCM and RM, whereas there was greater expression of M1 and M2 macrophage genes associated with RM not seen in CCM. Therefore, we hypothesise that although CCM possess fewer monocytes, because they differentiate to a predominately DC population, antigen presentation is more efficient and so a low M:L still induces T-cell activation. Whereas, RM possess more monocytes, but have a mixed population of DCs, M1 and M2 macrophages, which may interact and inhibit each other, leading to less efficient T-cell priming. The cause of this difference is unclear, but could be due to antigen load and disease burden, inducing more monocytes and differentiation to a wider variety of cell types in the RM population in comparison to CCM. However, we recognise that whilst this analysis has been standardised between the cohorts by interrogation of a uniform number of cells (1 × 10^6^ PBMCs) the gene expression profiles measured may be influenced by the relative abundance of each cell subsets between individuals and across the species. Future studies could address this by isolating and applying gene expression analysis to monocyte and lymphocyte cell populations in isolation. Within the two populations, differences in gene expression were also seen between progressors and controllers, suggesting that there could be a gene signature associated with protection which should be investigated further in future work.

Future work will involve characterisation of the T-cell response following infection, and the direct measurement of the number of macrophages and DCs using flow cytometry. A deeper understanding of the CCM’s natural ability to control TB could provide essential information for development of novel therapeutics. In contrast, the RM model provides the opportunity to investigate approaches to reduce the M:L and determine the subsequent impact on their ability to control TB. Macaque models that are able to represent different human populations, such as those with either a high or low risk of developing TB are essential as newly developed therapeutics may have different effects in these populations.

## Methods

### Experimental animals and MTB infection

The rhesus macaques (RM) and cynomolgus macaques were obtained from established breeding colonies in the United Kingdom (MCM, RM) and China (CCM). Genetic analysis of macaques from the UK colonies has previously confirmed the rhesus macaques to be of the Indian genotype and cynomolgus macaques of Mauritian genotype. All animals were between 2 and 4 years old at the time of exposure to *M. tuberculosis* and were naïve prior exposure to mycobacterial antigens (*M. tuberculosis* infection or environmental mycobacteria), as demonstrated by a negative tuberculin test while in their original breeding colony and by the IFNγ-based Primagam test kit (Biocor; CSL, Kansas, US) or, screening using an *ex-vivo* IFNγ ELISPOT (MabTech, Nacka, Sweden) to measure responses to mycobacterial antigens: purified protein derivative (PPD) batch RT50 (Statens Serum Institut (SSI), Copenhagen, Denmark), and 15-mer peptide pools of ESAT-6 and CFP-10 (Peptide Protein Research Ltd., Fareham, U.K.) just prior to the start of the study. Following exposure to MTB, macaques were housed in facilities compliant with the Advisory Committee for Dangerous Pathogens Level 3 (ACDP3) regulations.

Animals were housed in compatible social groups, in accordance with the Home Office (UK) Code of Practice for the Housing and Care of Animals Used in Scientific Procedures (1989), and the National Committee for Refinement, Reduction and Replacement (NC3Rs) Guidelines on Primate Accommodation, Care and Use, August 2006. Animals were sedated by intramuscular (IM) injection of ketamine hydrochloride (Ketaset, 100 mg/ml, Fort Dodge Animal Health Ltd, Southampton, UK; 10 mg/kg) for procedures requiring removal from their housing. None of the animals had been used previously for experimental procedures and each socially compatible group was randomly assigned to a particular study treatment. All animal procedures and study design were approved by the Public Health England, Animal Welfare and Ethical Review Body, Porton Down, UK, and authorised under an appropriate UK Home Office project license.

### ***M. tuberculosis*** challenge strain and aerosol exposure

The preparation of the Erdman K01 stock (HPA-Sept 2011) used for challenge and the methodology and apparatus used to deliver MTB via the aerosol route was as previously described [11]. In brief, mono-dispersed bacteria in particles were generated using a 3-jet Collison nebuliser (BGI, Butler, NJ, USA) and, in conjunction with a modified Henderson apparatus [17], delivered to the nares of each sedated primate via a modified veterinary anaesthesia mask. Challenge was performed on sedated animals placed within a ‘head-out’, plethysmography chamber (Buxco, Wilmington, NC, USA) to enable the aerosol to be delivered simultaneously with the measurement of respired volume [11, 18].

### Clinical procedures

Animals were monitored daily for behavioural abnormalities including depression, withdrawal from the group, aggression, and clinical changes in feeding patterns, respiration rate and coughing. On each occasion that required blood sample collection, aerosol challenge or euthanasia, animals were weighed, rectal temperature measured and examined for abnormalities. Red blood cell (RBC) haemoglobin levels were measured using a HaemaCue haemoglobinometer (Haemacue Ltd, Dronfield, UK) to identify the presence of anaemia, and erythrocyte sedimentation rates (ESR) were measured using the Sediplast system (Guest Medical, Edenbridge, UK) to detect and monitor inflammation induced by infection with MTB.

The time of necropsy, if prior to the end of the planned study period, was determined by experienced primatology staff and based on a combination of the following adverse indicators: depression or withdrawn behaviour, abnormal respiration (dyspnoea), loss of 20% of peak post-challenge weight, ESR levels elevated above normal (>20 mm), haemoglobin level below normal limits (<100 g/dL), raised temperature (>41 °C) and abnormal thoracic radiograph.

### Disease burden measures

Disease burden was measured using the approach described previously [41]. In brief, a post-mortem examination was performed immediately following euthanasia and pathological changes were scored using an established system based on the number and extent of lesions present in the lungs, spleen, liver, kidney and lymph nodes. Following fixation, magnetic resonance (MR) images of the lungs were collected and lesions identified based on their signal intensity and nodular morphology relative to more normal lung parenchyma. The total lung and lesion volume relative to the fixed tissue was determined using the Cavalieri method applied to MRI image stacks, and then expressed as a ratio to provide a measure of disease burden in each animal, as previously described [31,32]. Subsequently, the fixed lungs were sliced serially and lesions counted [35]. Animals which developed levels of disease that met humane endpoint criteria within 12 weeks of aerosol exposure to *M. tuberculosis* requiring the animals to be removed from further study and euthanized, were classified as ‘Progressors’. Animals in which the level of disease remained below humane endpoint criteria during the first 12 weeks after exposure to *M. tuberculosis*, were classified as ‘Controllers’.

### HEMAVET haematology analyser

The HEMAVET HV950FS analyser (Drew Scientific, USA) was operated according to the manufacturer’s instructions, using 20 µl blood collected from NHPs mixed with the supplied diluent (MULTI-CELL3TM DILUENT) which contains agents to lyse the erythrocytes and stabilise the haemoglobin for measurement^15^. Data was collected using this method for 10 RM and 9 CCM pre-infection.

### IDEXX Lasercyte Haematology analyser

The IDEXX Lasercyte analyser (IDEXX, USA) was used according to the manufacturer’s instructions using 500 µl of whole blood anticoagulated with EDTA (1.8 mg/ml of blood), in a Vacutainer (BD Biosciences, USA). Data was collected using this method for 18 CCM and 12 MCM pre-infection.

### Whole blood flow cytometry (FC) assay

Fifty microlitres of whole blood was collected and anticoagulated using heparin and stained for CD3-AF700, CD4-PeCy7 and CD8-PE (all Becton Dickinson, USA) and erythrocytes were lysed using Utilyse reagents (Agilent Technologies Inc, USA) and Flow Count Fluorosphere (Beckman Coulter, USA) beads were added to enable cell population quantification. Samples were analysed on a Beckman Coulter Cytomics FC500 flow cytometer (Beckman Coulter, USA) and data was analysed using CXP analysis V 2.0, 2.1 and 2.2 (Beckman Coulter, USA) Lymphocytes and monocytes were gated using forward and side scatter characteristics. Pre-infection, 9 CCM, 12 RM and 9 MCM had data collected using whole blood flow cytometry. Post-infection, 7 CCM, 9 RM and 4 MCM had data generated using this method, and was carried out at ACDP3 level of containment.

### Peripheral Blood Mononuclear Cells (PBMC) flow cytometry assay

PBMCs were isolated from whole blood anticoagulated with heparin (132 Units per 8 ml blood) (BD Biosciences, USA) using standard methods. Of note is that the material used for density gradient centrifugation was adjusted dependent on the macaque species, with a Ficoll Histopaque gradient (GE Healthcare, USA) used with Rhesus macaque blood and a Percoll gradient (GE Healthcare, USA) used with cynomolgus macaques. PBMCs were stored at −180 °C until resuscitated for the flow cytometry assay. Samples were analysed on a BD Fortessa flow cytometer (Becton Dickinson, USA) and data was analysed using FlowJo software (FlowJo, USA). Lymphocytes and monocytes were gated using forward and side scatter characteristics. Data was collected using with method for 17 RM, 24 CCM and 23 MCM pre-infection.

### PPD-specific IFNγ SFU as measured using the ELISPOT assay

An IFNγ ELISpot assay was used to estimate the frequency and IFNγ production capacity of mycobacteria-specific T cells in PBMCs using a human/simian IFNγ kit (MabTech, Nacka. Sweden), as described previously^16^. ELISPOT data was obtained from 9 RM, 7 CCM and 4 MCM post-infection with MTB and the assays were carried out at ACDP3 level of containment.

### Microarray analysis

Standardised numbers of isolated PBMCs were cultured with and without 30 µg PPD for 24 hours and then RNA was extracted for analysis microarray as described previously and was carried out at ACDP3 level of containment^17^. Briefly, RNA was isolated using Trizol (Invitrogen, Paisley UK) according to the manufacturer’s instructions. RNA was purified using RNeasy spin columns (Qiagen, Crawley, UK) with an on-column DNA digest step (Qiagen, Crawley, UK). RNA was quantified using a NanoDrop ND1000 UV-vis spectrophotometer (NanoDrop Technologies, Cambridge, UK) and the quality assured using Agilent 2100 Bioanalyzer (Agilent Technologies, Stockport, UK). All samples had an RNA Integrity Number (RIN) of at least 7.0. 50 ng of RNA was used for microarray analysis and was amplified and labelled using the Agilent Low Input Quick Amplification Labelling kit (Agilent Technologies, Stockport, UK) with RNA spike-ins as per the manufacturer’s instructions. Hybridisation was performed as per the Agilent 60-mer oligo microarray protocol using the Agilent Gene Expression Hybridisation kit. Data analysis was carried out using GeneSpring GX12 (Agilent Technologies, Stockport, UK) and data underwent quantile normalisation and expression profiles post-challenge were baseline transformed to expression at naïve time points for each animal. Genes were filtered according to expression and fold change so only genes that were detected in over 50% of samples and had a fold change of 2 were used for further analysis. A moderated T-test with Benjamin-Hochberg false discovery rate (FDR) correction for multiple testing was performed to identify differentially expression gene with a corrected p-value threshold of 0.01. The CCM microarray data is available at the Gene Expression Omnibus (GEO) database under the accession number GSE42273. The RM microarray data is available at the ArrayExpress database under the accession number E-MTAB-7558.

### Statistical analysis

**Figure 6:**
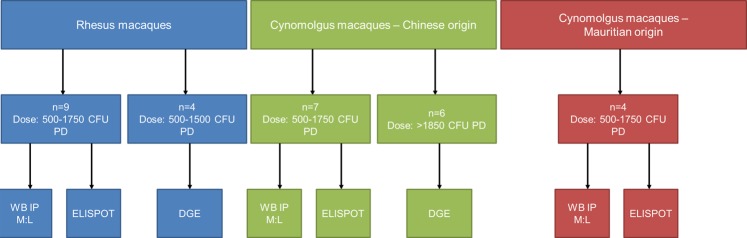
Diagram demonstrating the work conducted with each of the three macaque populations showing the number of individuals exposed to each aerosol dose and the subsequent analysis performed post MTB infection.

The data for these analyses were collected from *M. tuberculosis* infected macaques enrolled in several studies performed at PHE Porton to establish new aerosol challenge models for the evaluation of new TB therapeutics. These studies aimed to evaluate and characterise; the immunogenicity and efficacy BCG, and the outcome of infection with *M. tuberculosis* in rhesus and cynomolgus macaque species. Data were combined for each analysis from similarly treated individuals across studies i.e. same time after exposure, to the same strain and dose of *M. tuberculosis*. The data available for each assessment of the M:L ratio is shown in Table 1 and a work flow of animals from each study that had analysis applied post challenge is presented in Fig. 6. Differences between M:L ratios were evaluated using GraphPad Prism software v6 (GraphPad Software Inc, USA). Mann-Whitney U non-parametric tests were carried out on pre and post-infection M:L data. A log-rank Mantel-Cox test was carried out on the survival curves. The null hypothesis for the investigation was that there would be no differences between the populations in terms of M:L and other immune parameters and the alternative hypothesis was that those that were able to control MTB would have a different immune profile.
